# Lavender oil preparation Silexan is effective in mild-to-moderate major depression: a randomized, placebo- and reference-controlled trial

**DOI:** 10.1007/s00406-024-01783-2

**Published:** 2024-04-01

**Authors:** Siegfried Kasper, Hans-Peter Volz, Hans-Jürgen Möller, Sandra Schläfke, Stephan Klement, Ion-George Anghelescu, Erich Seifritz

**Affiliations:** 1 Center for Brain Research, Medical University of Vienna, Spitalgasse 4, 1090 Vienna, Austria; 2 Würzburg, Former Medical Director Hospital for Psychiatry, Psychotherapy and Psychosomatic Medicine Schloss Werneck, Werneck, Germany; 3 Clinic and Policlinic for Psychiatry and Psychotherapy, Ludwig Maximilian University, Munich, Germany; 4 Department of Research and Development, Dr. Willmar Schwabe GmbH & Co. KG, Karlsruhe, Germany; 5 Clinic of Psychiatry and Psychotherapy, Mental Health Institute Berlin, Berlin, Germany; 6 Department of Psychiatry, Psychotherapy and Psychosomatics, Psychiatric Hospital, University of Zürich, Zürich, Switzerland

**Keywords:** Silexan, Lavender, Depression, Clinical trial, Treatment efficacy

## Abstract

Anxiety and depressive disorders have overlapping symptoms and share common neurobiological pathways. Antidepressant drugs have been demonstrated to be efficacious in anxiety as well. Vice versa, it may also be promising to investigate the efficacy of anxiolytic drugs such as silexan in major depressive disorder (MDD). Patients with a mild or moderate, single or recurrent episode of MDD and a total score of 19–34 points on the Montgomery Åsberg Depression Rating Scale (MADRS) were randomized to receive 1 × 80 mg/d silexan, 1 × 50 mg/d sertraline, or placebo double-blind, double-dummy for 56 days. The primary outcome measure was the MADRS total score change between baseline and treatment end. Treatment groups were compared using a treatment policy estimand. 498 subjects (silexan 170, sertraline 171, placebo 157) were treated and analyzed. After 8 weeks, silexan and sertraline were superior to placebo for MADRS total score reduction, with absolute differences to placebo of 2.17 (95% confidence interval: 0.58; 3.76) points and 2.59 (1.02; 4.17) points, respectively (*p* < 0.01). Moreover, silexan was superior to placebo for alleviation of functional impairment according to the Sheehan Disability Scale with a difference of 2.40 (1.04; 3.76) points (*p* < 0.001). Both treatments were well tolerated; eructation was the most frequent adverse effect of silexan. The study confirms the antidepressant efficacy of silexan in mild or moderate MDD, including significant improvements in the subjects’ functional capacity. The results for sertraline confirm the assay sensitivity of the trial. Both drugs were well tolerated.

Trial registration

EudraCT2020-000688–22 first entered on 12/08/2020.

## Introduction

Silexan^1^ is an essential oil for oral administration produced from *Lavandula angustifolia* flowers that is registered as a medicinal product for patients suffering from anxiety disorders. The efficacy of silexan in subthreshold and generalized anxiety disorder as well as in mixed anxiety and depressive disorder (MADD) has been demonstrated in randomized, double-blind, placebo- and reference-controlled clinical trials and has been the subject of several reviews [1–11].

Anxiety disorders and depression are highly comorbid [12, 13], and anxiety disorders have been shown to predict later depression and vice versa [14]. Both conditions also show overlapping symptoms including anhedonia, sad mood, and worry [15] and neurobiological alterations [16]. It is, therefore, not surprising that antidepressant drugs such as selective serotonin reuptake inhibitors (SSRIs) and selective norepinephrine reuptake inhibitors (SNRIs) also show efficacy and are recommended as first line treatment for anxiety disorders [17]. Vice versa, it may also be promising to investigate potential antidepressant effects of compounds originally authorized for the treatment of anxiety.

Studies in which depression scales were administered as secondary or co-primary endpoints in patients with anxiety disorders consistently indicate that silexan, administered at the marketed dose of 1 × 80 mg/day, may also have an antidepressant effect. A recent meta-analysis of five clinical trials [11] showed significant superiority of silexan over placebo for the score reduction of item ‘Depressed mood’ of the Hamilton Anxiety Rating Scale (HAMA; available for all studies) as well as for the standardized total scores of the Hamilton Depression Rating Scale (HAMD) or the Montgomery-Åsberg Depression Rating Scale (MADRS) (available for three studies). Effect sizes favoring silexan were larger in a subset of patients who were at least mildly depressed at baseline. In a study in MADD [10], subjects treated with 1 × 80 mg/d Silexan for 10 weeks showed significantly more pronounced total score reductions for the HAMA and for the MADRS; [18] than those who received placebo and also showed more pronounced improvements of impaired daily living skills and health-related quality of life. Two working groups reviewed randomized, controlled trials where various preparations from *Lavandula angustifolia* were administered to patients with depressive disorders [19, 20]. Studies included investigations of oral preparations (including silexan), aromatherapy, aroma massage, and dermal presentations. The authors of both reviews concluded that the overall results indicated a clear antidepressant effect but that the studies were partly small, had heterogeneous patient selection criteria, investigated heterogeneous treatments, and thus needed confirmation in adequately sized, well-designed trials. A meta-analysis of randomized, controlled trials with different lavender preparations and routes of administration was published by another working group [21]. A significant antidepressant effect of lavender was found in seven out of ten eligible trials and for all trials combined.

An antidepressant effect of silexan could be explained by its pharmacological profile [22, 23]. In short, silexan was shown to enhance several parameters of neuroplasticity in cell and in-vivo models. Friedland et al. [24] observed an upregulation of the neuronal marker protein GAP-43 and a significant increase of neurite outgrowth by silexan. Moreover, the authors reported a beneficial effect on synaptogenesis already at low concentrations of silexan. In another study [25], inhalation of a commercially available lavender oil enhanced neuronal proliferation and dendritic complexity in the hippocampus and the supraventricular zone and elevated the serum levels of the neurotrophic factor BDNF. The findings indicate a beneficial effect of lavender oil on neuroplasticity and neurogenesis, which appears to be a final common pathway of antidepressant drugs in general [22]. Silexan causes a significant increase in the density of 5-HT_1A_ receptors and reduces their binding potential, which leads to increases in extracellular serotonin, dopamine, and norepinephrine [26, 27], a mechanism typically observed in serotonergic antidepressants. Moreover, lavender oil was shown to reduce corticosterone-induced increase in immobility times and thus to improve depression-like behavior [25]. While corticosterone treatment led to a reduction of the number of bromodeoxyuridine-positive cells in the hippocampus and the subventricular zone and to a reduced dendritic complexity of immature neurons, the contrary was observed during lavender oil co-administration. The anxiolytic effect of silexan was found to be related to an inhibition of the voltage-dependent calcium channels in synaptosomes, primary hippocampal neurons, and stably overexpressing cell lines [28], which caused an attenuation of the overreaching, situationally inadequate stress response of the central nervous system associated with anxiety and depressive disorders (e. g., [29]).

Following a series of clinical studies investigating the effects of silexan on depression secondary to or co-occurring with anxiety, this work presents the results of the first randomized, placebo- and reference-controlled clinical trial of silexan in patients suffering from an episode of mild-to-moderate major depressive disorder (LEAD study).

## Methods

### Objectives, design overview, ethics

This double-blind, double-dummy, randomized, controlled parallel-group study was performed with the primary objective of demonstrating the antidepressant efficacy of silexan by showing superiority over placebo. The SSRI sertraline was included as an active control for demonstrating the assay sensitivity of the trial.

Participants underwent a 3–7 days treatment-free screening period after which eligible subjects were randomized to double-blind treatment with silexan, sertraline, or placebo at a ratio of 1:1:1 (baseline visit). After 56 days of randomized treatment, a 7-day follow-up phase was observed for the down-titration of sertraline in order to minimize the risk of SSRI discontinuation symptoms [30], in which the subjects from the other groups received placebo. Participants were assessed for treatment efficacy and safety at 1, 2, 4, 6, and 8 weeks after baseline, and for safety at the end of the follow-up. In accordance with the estimands framework of the trial, subjects terminating treatment prematurely were asked to continue participating in the study procedures until the scheduled end. In case of early termination of the trial participation, examinations scheduled at week 8 were to be performed unless subjects were lost to follow-up or revoked their informed consent.

The study protocol was reviewed and approved by the competent independent ethical committees. All subjects provided written informed consent to participate and for the processing of their data. The principles of Good Clinical Practice and the Declaration of Helsinki were adhered to.

### Participants

Adult male or female out-patients of any ethnic group suffering from a mild-to-moderate, single or recurrent episode of major depressive disorder (MDD) meeting the diagnostic criteria of ICD-10 categories F32.0, F32.1, F33.0, or F33.1 [31] were screened for participation. The diagnostic procedures included the administration of the Mini-International Neuropsychiatric Interview [M.I.N.I.; 32], which the participating investigators had been trained to perform and interpret before assessing study participants. Eligible subjects also had to have a MADRS total score between 19 and 34 points at both screening and baseline and had to undergo treatment by a general practitioner or by a psychiatrist.

Main specific exclusion criteria were a diagnosis of severe MDD, any clinically important psychiatric or neurological diagnosis other than the study indication within 6 months before enrolment, administration of any psychotropic drugs, intravenous methylene blue, or linezolid within 30 days, or psychotherapy within 2 weeks before randomization. Subjects with a score ≥ 1 point for the Beck Scale for Suicide Ideation 5-Item Screen [33] or ≥ 1 point for MADRS item ‘Suicidal thoughts’ were excluded; both criteria had to be re-evaluated at each post-baseline visit. Subjects who had not responded to adequate antidepressant pharmacotherapy in the current episode of depression or who had failed to respond to ≥ 50 mg/day sertraline given for at least 6 weeks in any previous episode were also not randomized. Concomitant psychotropic medication and psychotherapy were not allowed during study participation.

### Interventions, blinding

Silexan is a proprietary essential oil produced from *Lavandula angustifolia* flowers by steam distillation and complies with the monograph Lavender oil of the European Pharmacopoeia [Ph. Eur.; 34]. It exceeds the quality requirements of the Ph. Eur. monograph on lavender oil. Batch to batch consistency is assured by a well-defined, standardized manufacturing process. Immediate release soft capsules containing 80 mg of silexan were used. Sertraline, the active comparator, is an SSRI that is recommended by current disease management guidelines as first-line treatment of major depressive disorders (e. g., [35, 36]). Film-coated tablets containing 50 mg of sertraline were obtained as merchandize and were packed in capsules. For both silexan and sertraline, identically matched placebo capsules were available. The smell of the investigational treatments was matched by flavoring the capsules containing silexan placebo with 1/1000 of the amount of lavender oil contained in the Silexan capsules.

Randomized subjects had to take 1 capsule each of the assigned active treatment and of placebo (i. e., silexan plus sertraline placebo, sertraline plus silexan placebo, or silexan placebo plus sertraline placebo), unchewed in the morning of each day of the randomized treatment phase. The daily dose of silexan was established in accordance with the existing marketing authorization of the product. For sertraline, reviews have shown that a ‘standard’ dose of 50 mg/d is sufficient for the treatment of mild-to-moderate MDD in the majority of cases, and that a ceiling effect for efficacy occurs already at the ‘standard’ dose (e. g., [37]), whereas a dose increase beyond the ‘standard’ dose may lead to a significant increase of treatment withdrawals due to adverse effects [38].

### Measures of efficacy and safety

The primary outcome measure for treatment efficacy was the absolute intraindividual change of the observer-rated MADRS total score between baseline and the final examination at week 8. Uniformity of assessments was assured by performing a mandatory rater training (including a performance review with determination of the inter-rater reliability) before the start of patient recruitment. Rates of response (a total score decrease by at least 50% of the baseline value) and remission (a total score of < 10 points) at treatment end were pre-defined as secondary efficacy outcomes. Further standardized scales assessed as secondary efficacy outcome measures included the self-rated, revised Beck Depression Inventory [BDI-II; 39], the nine-item Depression module of the self-rated Patient Health Questionnaire (PHQ-9); [33], the observer-rated Clinical Global Impressions scale (CGI); [40], as well as the Sheehan Disability Scale (SDS); [41], which is a tool for brief self-rating of the disease-associated, functional disability regarding work, social life, and family relationships. Safety and tolerability outcomes were treatment discontinuations due to lack of efficacy or tolerability, adverse events (AEs; including those reported during the down-titration phase), physical and ECG examinations, vital signs, and routine laboratory measures.

### Random sequence generation, allocation concealment, implementation

The random code was generated by a biostatistician of the contract research organization that performed the trial who was otherwise not involved in the project. A validated SAS macro was used for generating the random code. Treatments were randomized in fixed-size blocks (ratio: 1:1:1) with stratification by site. The block size was withheld from the investigators until unblinding in order to reduce the predictability of the treatments.

The study drugs were dispensed to the sites in numbered containers. The investigators were instructed to assign the lowest available random number after confirming a patient’s eligibility.

### Statistical methods, sample size

The analysis of the study was planned and performed using an estimands framework. The primary estimand used during confirmatory testing of the treatment group difference between Silexan and placebo for the change in the MADRS total score between baseline and week 8 was mainly based on a treatment policy strategy according to which intercurrent events (prohibited concomitant treatment, treatment non-compliance, premature treatment discontinuation without subsequent prohibited antidepressant therapy) were ignored and the outcomes data were analyzed as observed. Missing data were multiply imputed using only data from the placebo group. For subjects switched to an alternative antidepressant therapy after premature treatment discontinuation, outcomes data obtained after the switch were ignored, and the resulting missing values were multiply imputed using only data from the placebo group (hypothetical strategy). Secondary efficacy outcomes, including the comparisons between sertraline and placebo and between Silexan and sertraline, were tested descriptively using an analogous estimand.

Analyses of primary and secondary efficacy outcomes were performed on the full analysis set (FAS), which included all subjects who had received the randomized treatment at least once. A per-protocol analysis as well as analyses using different estimands and data imputation strategies were included as sensitivity analyses. Subject eligibility for the different analysis data sets was determined before code breaking. For continuous outcomes, change from baseline was tested using analysis of covariance (ANCOVA) on the imputed data sets, with factors for treatment and site, and the baseline value as a covariate. Overall adjusted treatment group differences were estimated by applying Rubin’s rules to the estimates from the imputed data sets [42]. Treatment effect estimates for the comparisons of Silexan and sertraline with placebo were obtained in separate ANCOVA models, and hence the adjusted mean value and associated variance estimates for the placebo group in both comparisons are numerically not identical even though the same subjects were analyzed. Differences between proportions were tested using *χ*^2^-tests and logistic regression analysis. For the confirmatory test of the primary outcome, a one-sided type I error level of *α* = 0.025 was applied. All other *p*-values are two-sided unless otherwise noted; two-sided *p*-values ≤ 0.05 are considered descriptively significant for illustrative purposes, but should not be interpreted as proof of treatment effects in the underlying patient population. The results presented below apply to the estimand in the FAS unless otherwise noted.

According to the literature, a MADRS total score between-group mean value difference of 2 points is considered clinically meaningful (e. g., [43]). For the sample size calculation, we expected a difference of 3 points and a common standard deviation of 8 points based on previous studies with Silexan. Under these assumptions, a sample size of at least 151 subjects per group provides a power of 90% for rejecting the null hypothesis using a one-sided type I error level of *α* = 0.025 (*t*-test).

## Results

### Recruitment, participant flow

**Fig. 1:**
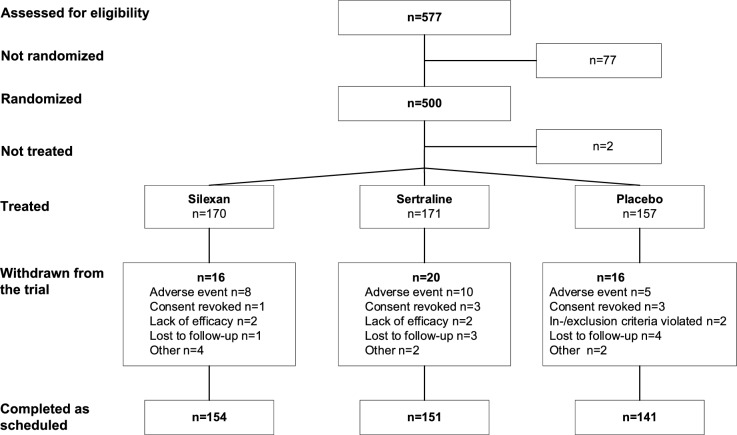
Disposition of subjects, analysis data sets

The trial was conducted between October 2020 and April 2023 in 53 general and psychiatric or neurological practices in Germany and Poland. A total of 577 subjects were screened for eligibility. Of these, 500 subjects were randomized, 498 were treated, and 446 (90.6% of 500) completed the study as scheduled (Fig. 1). Two subjects who were randomized but not treated had been assigned to placebo; one was lost to follow-up and the other revoked the informed consent. Withdrawal rates were similar in all treatment groups. During randomized treatment, adverse events were the most frequently reported reason for premature withdrawal from the trial in all groups.

Participants who had been randomized but not treated were excluded from all analyses, and thus 498 subjects (silexan 170/sertraline 171/placebo 157) were analyzed for safety as well as for efficacy in the FAS. A total of 434 subjects (silexan 149/sertraline 147/placebo 138) completed the study without major protocol deviations and were thus eligible for the per-protocol (PP) analysis.

Twenty-one subjects in the silexan group (12.4%), 24 in the sertraline group (14.0%), and 19 in the placebo group (12.1%) had at least one intercurrent event. The most frequent event was premature discontinuation of the investigational treatment (silexan 19/sertraline 22/placebo 18), followed by initiation or adjustment of prohibited medication (2/2/0) and treatment non-compliance (intake < 80% or > 120%; 1/2/1).

### Baseline data and treatment compliance

Main demographic and baseline characteristics of the full analysis set are shown in Table 1. About ^2^/_3_ of the participants in all groups were female. All subjects except five were Caucasians. According to ICD-10 diagnostic categories, 230 subjects (46.2%) suffered from a mild and 268 (53.8%) from a moderate episode of MDD. Two hundred and sixteen participants (43.4%) were treated for their first depressive episode while 282 (56.6%) had a recurrent episode, with a median number of three previous episodes for all groups. At inclusion, the average duration of the current episode ranged between 0 and 11 months (mean ± SD: 3.4 ± 2.8 months).

**Table 1:** Demographic data and efficacy outcome measures at baseline (full analysis set; absolute frequency and % or mean ± SD and range)

	Silexan (*n* = 170)	Sertraline (*n* = 171)	Placebo (*n* = 157)
Sex			
Female	118 (69.4%)	110 (64.3%)	99 (63.1%)
Male	52 (30.6%)	61 (35.7%)	58 (36.9%)
Age (years)	45.8 ± 14.5 18–83	44.7 ± 14.4 10–84	46.6 ± 15.7 18–82
Characteristics of current episode of depression			
Mild	79 (46.2%)	83 (48.8%)	68 (43.3%)
Moderate	87 (51.2%)	92 (53.8%)	89 (56.7%)
First episode	71 (41.8%)	76 (44.4%)	69 (43.9%)
Recurrent episode	99 (58.2%)	95 (55.6%)	88 (56.1%)
Duration of current episode at inclusion (months)	3.3 ± 2.8 0–11	3.5 ± 2.9 0–11	3.4 ± 2.7 0–11
MADRS total score	23.3 ± 3.5 19–34	23.6 ± 3.4 19–34	23.4 ± 3.4 19–34
BDI-II total score	21.3 ± 8.3 0–46	21.5 ± 8.9 1–46	21.7 ± 8.5 1–45
CGI Item 1 (Severity of Illness)			
≤ mildly ill	54 (31.8%)	46 (26.9%)	51 (32.5%)
≥ moderately ill	116 (68.2%)	125 (73.1%)	106 (67.5%)
mean ± SD	3.8 ± 0.7	3.9 ± 0.7	3.8 ± 0.7
PHQ-9 total score	11.7 ± 4.1 3–22	11.4 ± 3.9 2–20	11.4 ± 3.9 3–23
SDS	14.4 ± 6.4 0–30	13.9 ± 6.5 0–30	14.2 ± 6.4 0–28

*MADRS* Montgomery-Åsberg depression rating scale; *BDI-II* Beck depression inventory, *CGI* clinical global impressions scale, *PHQ-9* patient health questionnaire, total score, *SDS* Sheehan disability scale, global impairment score

At baseline, co-existing conditions that required pharmacological treatment were reported by 50.6% of the subjects in the silexan group, by 36.8% in the sertraline group, and by 42.0% in the placebo group.

The treatment groups were well balanced with respect to the baseline values of all efficacy outcomes (Table 1).

Based on tablet counting, the average treatment compliance for the period between baseline and the week 8 visit was 99.1% ± 6.4% for silexan, 98.6% ± 4.9% for sertraline, and 99.1% ± 5.7% for placebo (compliance outside a range of 80–120% was accounted for as an intercurrent event).

### Efficacy

**Fig. 2:**
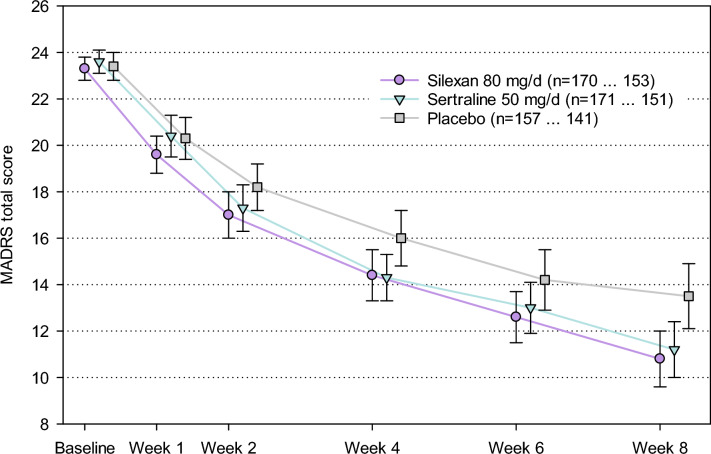
Montgomery-Åsberg Depression Rating Scale (MADRS) total score (means and 95% confidence intervals; primary estimand without imputation of missing values, full analysis set; legend shows numbers of subjects with valid data at baseline and week 8; *p* < 0.01 for change from baseline to week 8 for silexan vs. placebo and for sertraline vs. placebo)

The average MADRS total score decreased monotonically in all treatment groups until the end of the efficacy assessments at week 8 (Fig. 2). Total score decline in the silexan and sertraline groups was similar and more pronounced than in the placebo group. Based on average intraindividual differences to baseline, silexan was significantly superior to placebo by a margin of 2.17 points after the 8 weeks’ treatment (point estimate; *p* < 0.01; confirmatory test of the primary efficacy outcome measure; Table 2). Significant superiority over placebo was also observed for sertraline, supporting the assay sensitivity of the trial.

**Table 2:** Montgomery-Åsberg Depression Rating Scale—adjusted treatment group mean value differences for total score change from baseline to week 8 (primary estimand, full analysis set)

Comparison	Active treatment mean, 95% CI	Placebo mean, 95% CI	Adjusted mean value difference, 95% CI	*p*
Silexan vs. placebo	−12.10 [−13.30; 11.00]	−9.95 [−11.10; −8.77]	−2.17 [−3.76; −0.58]	0.008
Sertraline vs. placebo	−12.60 [−13.7; −11.50]	−10.02 [−11.19; −8.85]	−2.59 [−4.17; −1.02]	0.001

*CI* confidence interval

**Fig. 3:**
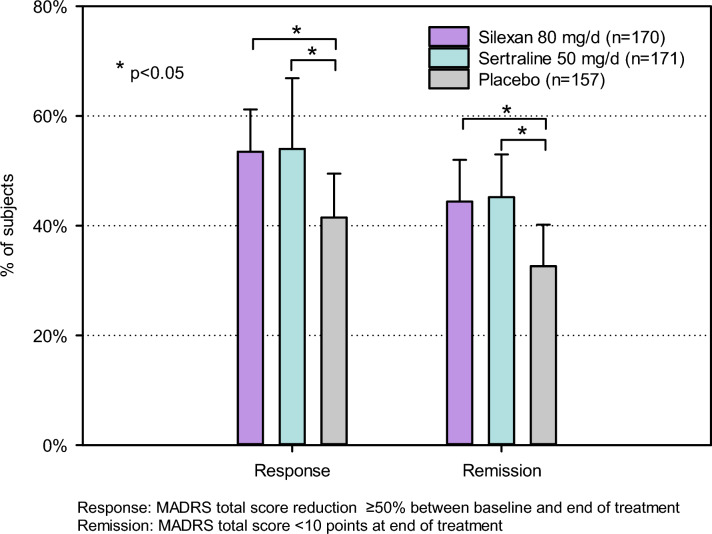
Treatment response and remission based on the Montgomery-Åsberg Depression Rating Scale (MADRS) total score (proportions and upper limits of 95% confidence intervals; primary estimand, full analysis set)

The main results are reflected in the proportions of responders and remitters (Fig. 3): while 53.5% and 54.0% of the subjects in the silexan group and in the sertraline group, respectively, showed a MADRS total score decrease from baseline by at least 50% and 44.4% and 45.2% had a total score below 10 points at treatment end, respectively, 41.5% of subjects randomized to placebo were responders and 32.6% were in remission. For both outcomes, differences in proportions between silexan and sertraline on one hand and placebo on the other were descriptively significant (*p* < 0.05). The associated numbers needed to treat (NNT) were 9 and 8 for response and also 9 and 8 for remission, for silexan and sertraline, respectively.

For the self-rated BDI-II and PHQ-9 depression scales, subjects treated with silexan showed clear symptom alleviation compared to baseline after eight weeks even though the treatment group difference did not reach the nominal level of statistical significance (Table 3). Compared to silexan-treated subjects, the average declines of the BDI-II and PHQ-9 total score in the placebo group were delayed by about 2 weeks (e. g., the decrease from baseline after 4 weeks’ treatment with placebo was comparable to the decrease after 2 weeks on silexan).

**Table 3:** Secondary efficacy outcomes—adjusted treatment group mean value differences for change from baseline to week 8 (primary estimand, full analysis set)

Comparison	Scale	Active treatment mean, 95% CI	Placebo mean, 95% CI	Adjusted mean value difference, 95% CI	*p*
Silexan vs. placebo	BDI-II	−10.06 [−11.36; −8.76]	−8.48 [−9.82; −7.13]	−1.58 [−3.38; 0.22]	0.085
	CGI-1	−0.95 [−1.11; −0.80]	−0.78 [−0.94; −0.62]	−0.18 [−0.39; 0.04]	0.111
	CGI-2	2.51 [2.32; 2.70]	2.72 [2.52; 2.91]	−0.21 [−0.47; 0.06]	0.127
	PHQ-9	−5.1 [−5.77; −4.42]	−4.26 [−4.95; −3.57]	−0.84 [−1.77; 0.09]	0.077
	SDS	−6.07 [−7.03; −5.10]	−3.67 [−4.68; −2.65]	−2.40 [−3.76; −1.04]	< 0.001
Sertraline vs. placebo	BDI-II	−10.84 [−12.13; −9.54]	−8.41 [−9.75; −7.07]	−2.43 [−4.23; −0.63]	0.008
	CGI-1	−1.04 [−1.19; −0.89]	−0.79 [−0.94; −0.63]	−0.25 [−0.46; −0.04]	0.020
	CGI-2	2.37 [2.18; 2.56]	2.74 [2.55; 2.94]	−0.37 [−0.63; −0.11]	0.006
	PHQ-9	−5.42 [−6.05; −4.79]	−4.18 [−4.83; −3.53]	−1.24 [−2.12; −0.36]	0.006
	SDS	−4.35 [−5.39; −3.31]	−3.54 [−4.60; −2.47]	−0.82 [−2.26; 0.63]	0.267

*CI* confidence interval, *BDI-II* Beck depression inventory (revised), total score, *CGI-1* clinical global impressions, item 1 (Severity of Illness), *CGI-2* clinical global impressions, item 2 (Global Change), *PHQ-9* patient health questionnaire, total score, *SDS* Sheehan disability scale, global impairment score

According to the CGI, the proportions of subjects who were at least moderately ill decreased from their baseline values (Table 1) to 21.8% (*n* = 37) for silexan, 22.8% (*n* = 39) for sertraline, and 32.5% (*n* = 51) for placebo at week 8 while 22 (12.9%), 21 (12.3%) and 14 subjects (8.9%), respectively, were assessed to be not ill at all. Eighty-one subjects in the silexan group (47.7%) were rated to be much or very much improved at treatment end, compared to 86 (50.3%) for sertraline and 63 (40.1%) for placebo.

For MDD-associated functional impairment, the results for the SDS shows descriptively significant improvements for silexan-treated subjects over the placebo group at week 8 (Table 3). In addition to the total score, significant mean value differences between silexan and placebo were also observed for each of the domains assessed by the SDS (work, social life/leisure activities, family life/home responsibilities; *p* ≤ 0.01). For sertraline, no significant SDS improvements over placebo could be observed.

### Safety/tolerability

The investigators observed 92 potentially treatment-related events in 56 subjects (32.9%) in the silexan group, 91 events in 49 subjects (28.7%) in the sertraline group, and 69 events in 43 subjects (27.4%) in the placebo group. Serious adverse events (any causal relationship) were observed in five subjects for silexan (2.9%), in six for sertraline (3.5%), and in four for placebo (2.6%). Two subjects in each group reported suicidal ideation. All other serious events occurred only once.

The most frequent potentially related events in the silexan group were eructation (28 subjects, 16.5%) and nasopharyngitis (6, 3.5%). For sertraline, the most frequent events were headache (11, 6.4%), nausea (8, 4.7%), and diarrhea (7, 4.1%). All other potentially related events were observed in less than 3% of subjects in the silexan or sertraline group.

Fourteen subjects in the silexan group (8.2%), 14 in the sertraline group (8.2%) and 7 in the placebo group (4,5%) terminated treatment prematurely for lack of efficacy or for intolerability. The results of the physical and ECG examinations, vital signs and routine laboratory measures were comparable in all treatment groups.

## Discussion

The results of the present randomized, controlled trial demonstrate that eight weeks’ treatment with 1 × 80 mg/d Silexan had a statistically significant and clinically relevant antidepressant effect in subjects suffering from a mild or moderate, single or recurrent episode of MDD. The observed average MADRS total score decrease from baseline (12.1 points after 8 weeks) as well as a minimum expected average improvement in the underlying patient population of eleven points (corresponding to the lower bound of the 95% confidence interval for the point estimate) were in the range of a clinically meaningful or clinically substantial change according to empirically derived criteria [44, 45]. For the comparison between Silexan and placebo, the observed mean value difference of 2.17 points for MADRS total score change exceeded the margin of two points that is considered clinically important [43, 46]. With a mean value difference to placebo of 2.59 points, sertraline also demonstrated a clear, statistically significant antidepressant effect. A comparison with the literature, e. g., the recent review and meta-analysis of Luo et al. [47] of the association between the dose of sertraline and the antidepressant response, in which more pronounced mean value differences vs. placebo of 4.15 points for MADRS total score change, 0.43 points for CGI Item 1, and 0.48 points for CGI Item 2 were observed, may nevertheless suggest that the present study may have tended to underestimate the true effects of the investigated antidepressant drugs. Moreover, the smaller absolute treatment effect of sertraline vs. placebo in the present study could also be related to the fact that our study included only subjects with mild or moderate depression, whereas the review of Luo et al. [47] also included studies with more severely depressed subjects. The same review found the dose–response curve for sertraline to be almost flat in a range between 50 and 150 mg/d so that an underdosing of the drug in the mildly to moderately depressed patient population of this trial appears to be unlikely. It can, however, also not be excluded that the sertraline treatment group may have included rapid metabolizers who may have had plasma levels below effective threshold. This is a potential limitation to the interpretation of our results on sertraline.

In a meta-analysis of the efficacy of ‘new-generation’ antidepressants [48], MADRS total score change mean value differences to placebo ranging between 1.06 and 4.59 points were reported for bupropion, duloxetine, escitalopram, paroxetine, venlafaxine, and vilazodone, with an over-all meta-analysis difference of 2.99 points. In comparison, the mean differences for both Silexan and sertraline determined in our study were slightly below average. While this could again be associated with the fact that the review of Hengartner et al. [48] considered studies into depression of any severity whereas our trial was performed in mild-to-moderate depression, it is also well known that an appreciable proportion of patients with depressive disorders do not respond to pharmacotherapy in controlled trials as well as in clinical practice, despite adequate dosing (e. g., [49, 50]). Seemingly modest between-group differences determined in an analysis of all treated participants of a depression trial may, therefore, ‘mask’ large, important differences observed in those who benefit from treatment [51].

Our results for the total scores of the self-rated depression scales BDI-II and PHQ-9 as well as for CGI items 1 and 2 consistently show advantages of Silexan over placebo and thus support the results for the MADRS even though the nominal level of statistical significance could not be achieved.

As a complement to the scales mentioned above, which focus on the clinical presentation of MDD, the SDS assesses the impact of the disorder on the patients’ functional abilities in daily living and thus represents a highly patient-relevant outcome. The observed average total score difference of 2.4 points between Silexan and placebo clearly exceeds the 2-point difference classified as clinically relevant by the authors of the scale [52]. It is important to note that significant improvements were observed for all functional areas of the SDS (work, social life/leisure activities, family life/home responsibilities), indicating that treatment with Silexan had an important beneficial effect on the subjects’ disease-associated quality of life. This is of particular importance as depression is widely recognized as a main cause of impaired quality of life and inability to work (e. g., [53]). For sertraline, comparable improvements of aspects of daily living could not be observed even though both treatments had similar antidepressant effects.

Adverse events in the Silexan group were mainly eructation, a known but mild and transient side effect that may be annoying but not harmful. This side effect might be mitigated or even avoided by simultaneous intake with a glass of water or ingestion just before a meal. The incidence rates of all other types of adverse events observed during treatment with Silexan were similar to those in the placebo group.

In conclusion, this first randomized, controlled trial with Silexan in patients with mild-to-moderate MDD demonstrates that this herbal medicinal product has a clinically meaningful, statistically significant antidepressant effect. For the primary outcome measure, relative MADRS total score change from baseline, the magnitude of the treatment effect was comparable to that of therapeutically dosed sertraline based on the comparisons between both treatments and placebo. Moreover, Silexan improved disease-associated functional impairment and quality of life. Silexan could thus complement the range of available therapeutic options for mild-to-moderate depression as a well-tolerated pharmacotherapeutic intervention with a different mode of action than established synthetic antidepressants.

## Data Availability

Raw data cannot be shared both due to ethical reasons and to data protection laws. To the extent permitted by law, the trial data required for validation purposes will be disclosed in result reports on corresponding databases. All relevant data are within the paper.

## Footnotes

1 Silexan® is the active substance of Lasea® (Dr. Willmar Schwabe GmbH & Co. KG, Karlsruhe, Germany).
